# Antiplatelet/anticoagulant agents for preventing thrombosis events in patients with severe COVID-19: A protocol for systematic review and meta-analysis

**DOI:** 10.1097/MD.0000000000021380

**Published:** 2020-08-07

**Authors:** Yiwei Li, Ying Xu, Pengfei Shi, Ying Zhu, Wei Hu, Can Chen

**Affiliations:** a Department of Intensive Care Unit; b Department of Hematology, Affiliated Hangzhou First People's Hospital, Zhejiang University School of Medicine, Zhejiang, PR. China.

**Keywords:** antiplatelet/anticoagulant, COVID-19, prophylaxis, thromboembolism

## Abstract

**Background:**

On March 11, 2020, World Health Organization announced that severe acute respiratory syndrome coronavirus 2 caused COVID-19 was a global pandemic. COVID-19 is associated with venous thromboembolism including deep vein thrombosis and pulmonary embolism. To further identify the current role of antiplatelet/anticoagulant therapy in the prophylaxis and treatment of COVID-19 patients is important.

**Methods:**

We will conduct a systematic review based on searches of major databases (eg, Pubmed, Web of Science, EMBASE, CENTRAL, MEDLINE, SCI-EXPANDED, CPCI-S, CBM, CNKI, and Wanfang Database) and clinical trial registries from inception to present without limitations of language and publication status. All published randomized control trials, quasi-randomized trials, retrospective and observational studies related to prophylactic antiplatelet/anticoagulant for severe COVID-19 will be included. Primary outcome includes incident acute thrombosis events. Second outcome is the incidence and severity of adverse effects. Full-text screening, data extraction and quality assessment will be conducted by 2 reviewers independently. The reporting quality, risk of bias, sensitivity analysis and subgroup analysis will be performed to ensure the reliability of our findings by other 2 researchers. The statistical analysis will be performed by RevMan V.5.3 software and Stata V.12.0 software.

**Results:**

The result of this systematic review will provide valid advice and consultation for clinicians on the management of prophylactic antiplatelet/anticoagulant for severe COVID-19 patients.

**Conclusion:**

This systematic review will provide evidence for prophylactic antiplatelet/anticoagulant of severe COVID-19 patients.

**PROSPERO registration:**

CRD42020186928.

## 1 Introduction

Severe acute respiratory syndrome coronavirus 2 (SARS-CoV-2) caused COVID-19 has been officially announced as a global pandemic by the World Health Organization since March 11, 2020. As of May 26, a total of 5495061 cases have been confirmed worldwide and 346232 deaths have been reported across 188 countries or regions. Currently, with over 1.3 million confirmed cases and over 82,000 deaths, the United States leads all countries.^[1]^ Most patients with COVID-19 presented mild illness, others requiring hospitalization may presented fatal critical illness. Of these severe patients, a high prevalence of acute cardiovascular events has been observed.^[2–7]^

SARS-CoV-2 and SARS-CoV have shared cellular target and some clinical manifestations, such as thrombocytopenia, prolonged thrombin time and elevated D-dimer levels.^[6,8]^ It means that similar with SARS-CoV, SARS-CoV-2 is likely to be complicated with coagulopathy namely disseminated intravascular coagulation (DIC) or pre-DIC secondary to an increased inflammatory state. Patient with DIC has a rather prothrombotic character with high risk of venous thromboembolism (VTE).^[8–10]^

Previous study reported that COVID-19 is associated with venous thrombotic events including deep vein thrombosis and pulmonary embolism (PE).^[11]^ Emerging evidence shows that with a profound hypercoagulable state, complicating VTE in patients with COVID-19 are common.^[12–14]^ The incidence of VTE in ICU patients without thromboprophylaxis was 25% with a mortality rate of 40%.^[15]^ The prevalence of VTE in the study of Cui et al seems to be higher than other studies including patients admitted in ICUs for other disease conditions.^[15,16]^ A meta-analysis indicated that patients with VTE in ICU had a marginally increased risk of in-hospital mortality (relative risk 1.31; 95% CI: 0.99–1.74).^[17]^ In a study of 449 patients with severe COVID-19, patients with markedly elevated D-dimer or sepsis-induced coagulopathy were benefit from anticoagulant therapy, especially low molecular weight heparin.^[18]^ Meanwhile, VTE is associated with infection. COVID-19, as a special infection, may has an increased VTE risk due to endothelial damage, microvascular thrombosis and occlusion, or even autoimmune mechanisms.^[19]^ Thus, it appears that severe COVID-19 patients are in the high risk of VTE and high rate of mortality, and prognosis may be improved by anticoagulant therapy.

However, patients with COVID-19 may have a prolonged activated partial-thromboplastin time (aPTT), which may indicate a clotting factor deficiency or the presence of an inhibitor.^[20]^ In the study of Bowles, 216 patients with severe SARS-CoV-2 were received for coagulation screening, and 44 (20%) presented with a prolonged aPTT, and most aPTT prolonged patients were positive for lupus anticoagulant (91%). No bleeding tendency has been observed.^[21]^ Thus, prolonged aPTT should not be a barrier to the use of thromboprophylaxis therapies with COVID-19. All these observations raise a challenging question that is what the current role of antiplatelet/anticoagulant therapy in the prophylaxis and treatment of COVID-19 patients is.^[22]^

## 2 Methods

### 2.1 Registration

**Figure 1:**
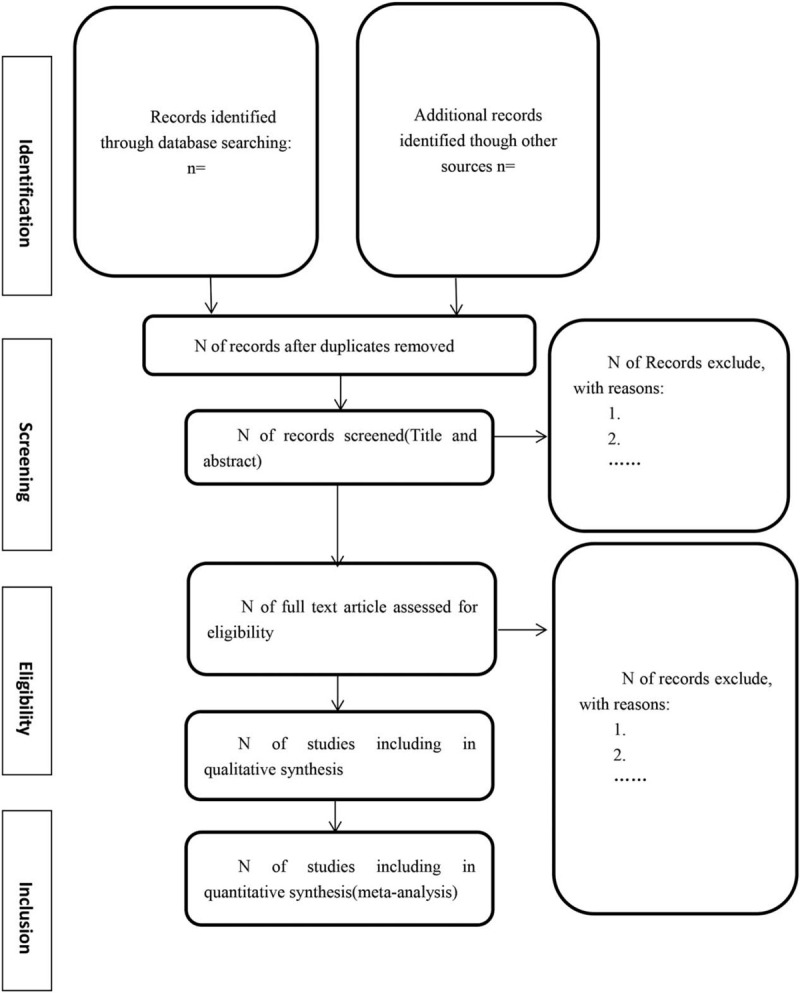
Flow diagram of literature search.

The protocol will be performed following the recommendations from the Cochrane Handbook of Systematic Review of Intervention and reported in accordance with the Preferred Reporting Items for Systematic Reviews and Meta-Analysis Protocols (PRISMA-P) statement (Fig. 1).^[23]^ It is registered in the PROSPERO database (CRD42020186928).

### 2.2 Eligibility criteria

Inclusion criteria followed the PICOS principles: patients, intervention, comparisons, outcome, and study design type.

#### 2.2.1 Type of participants

Gender, ethnicity, disease duration or ethnicity are not restricted for all participants diagnosed with severe pneumonia caused by COVID-19 according to the laboratory conformation (such as real-time PCR) and chest CT findings and required advanced life support.

#### 2.2.2 Type of comparisons and interventions

The aim of study is to assess the role of antiplatelet or anticoagulant as preventive agents for thrombosis events in patients with severe COVID-19, especially presenting with respiratory deterioration and/or hemodynamic instability. Therefore, patients will be assigned to intervention or control group. The intervention groups will include but not be limited to: antiplatelets (including aspirin, P2Y12 antagonists, αIIbβ3 antagonists), anticoagulants (heparin, vitamin K antagonists, direct thrombin inhibitors and direct factor Xa inhibitors), antiplatelets plus anticoagulants. The control group will include placebo or no thromboprophylaxis therapies at all. All therapeutic doses, the time of dosing, duration and means of administration (intravenous, oral) are no restricted.

#### 2.2.3 Type of outcome measures

The primary objective is the efficacy of antithrombotic treatment in preventing the first thrombosis. The primary outcomes are incident acute thrombosis (arterial or venous) events confirmed by appropriate imaging studies or death. The secondary outcomes are security index, which will be measured by the incidence and severity of adverse effects, including:

(1) bleeding events (significant bleeding events, clinically relevant insignificant bleeding events, or minor events).
(2) changes in aPTT, D-dimer and platelet levels.
(3) survival assays (main conclusions, mortality, survival time). Each adverse event will be separately assessed.

Thrombosis events were defined as 1 of the following events:

(1) deep vein thrombosis.
(2) PE.
(3) sudden death with no obvious cause (possible fatal PE).
(4) brain ischemia.
(5) acute myocardial infarction.

#### 2.2.4 Type of study

We will include randomized controlled trials, quasi-randomized trials, retrospective and observational studies, clinical trials and case series, irrespective of blinding, language, or publication status.

### 2.3 Exclusion criteria for study selection

We defined the following exclusion criteria:

(1) Studies in which the same patients have been enrolled.
(2) Commentaries, editorials, case reports, letters, editorials, and expert opinions.
(3) Missing or insufficient data that cannot be obtained after contacting original authors.
(4) Unpublished records such as conference papers, theses, and patent.
(5) Studies in which the patients are under 18 years old.

### 2.4 Methods for searching

#### 2.4.1 Electronic searched

2 reviewers will research Pubmed, Web of Science, Cochrane Central Register of Controlled Trials, Medical Literature Analysis and Retrieval System Online, Excerpa Medica database, Science Citation Index Expanded (SCI-EXPANDED), Conference Proceedings Citation Index-Science, Chinese Biomedical Literature Database, China Network Knowledge Information and Chinese Science Journal Database, Wanfang database (Wanfang Data) from their inception to the present. The full search strategy for Pubmed is provided in Table 1.

**Table 1:**
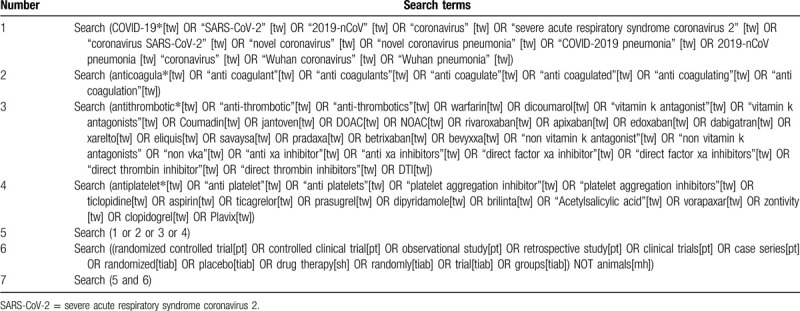
Search strategy for the PubMed database.

#### 2.4.2 Searching other resources

In addition, Reference lists of relevant trials and reviews will also be searched. For any unidentified clinical trials, we will search clinical trial registries, such as the International Clinical Trials Registry Platform (ICTRP), ClinicalTrials.gov, websites of US Food and Drug Administration (FDA), and European Medicines Agency (EMA) and contact the authors to receive the relevant data. We will also seek the COVID-19 Study Registry (https://covid-19.cochrane.org/ ) and COVID-evidence (https://covid-evidence.org/).

### 2.5 Study selection and data extraction

#### 2.5.1 Selection of data

The review will be analyzed by using Stata version 16 (StataCorp LLC, College Station, TX) and illustrated with the PRISMA statement.^[23]^ 2 reviewers will independently scan the title and abstract to remove obvious irrelevant studies. Where a study is potentially relevant, the full text will be retrieved and evaluate by 2 same reviewers to exclude the ineligible studies. Any discrepancy will be resolved through discussion or judged by a third reviewer.

#### 2.5.2 Data extraction and management

Relevant data will be extracted independently from included trials in a predefined form by 2 independent reviewers, which include the following information:

(1) The basic information of each study: first author, title, journal, time of publication, country, funding source, study design, and so on.
(2) characteristics of patients: average age, gender, past medical history, severity of COVID-19 pneumonia, comorbidity, and so on.
(3) interventions and comparators: types, doses, frequencies and lengths of thromboprophylaxis therapies and comparators where applicable, and so on.
(4) outcomes: measures, main conclusions, mortality, length of stay, rates and types of incident acute thrombosis (including arterial or venous) and adverse events, and so on.

In order to assume the integrity of extracted items, we will contact original authors via email to request for missing data or clarification necessary. If there is no response, with repeated contacts, the data will be reconsidered, or deleted if necessary. 2 reviewers will cross-check the received data and transfer into the Stata file. Disagreements will be judged by a third reviewer. Finally, the data will be recorded in a unified form.

### 2.6 Risk of bias assessment

As with the previous process, pilot risk of bias of the included studies will be assessed by 2 independent reviewers at study level in accordance with the guidance in the latest version of Cochrane Handbook for systematic reviews of interventions, which covers: sequence generation, allocation concealment, blinding of participants and personnel, blinding of outcome assessments, incomplete outcome reporting, selective reporting and other sources of bias.^[24]^ Each of the domains will be scored as “low risk” or “high risk” depending on the extracted information from each study. “Unclear” record will be made if there is insufficient detailed in the publication and will contact to authors of primary studies for missed or unpublished data. Disagreements will be judged by a third reviewer. The RevMan V5.5.3 software will be used to evaluate each study.

### 2.7 Data synthesis and statistical analysis

#### 2.7.1 Assessment of heterogeneity and data synthesis

We will analyze heterogeneity by considering the variability in the population characteristics, interventions and outcomes among included trials using RevMan V.5.3 software and Stata V.12.0 software. Dichotomous outcomes such as occurrence of thrombosis events will be determined by using risk ratios with a confidence interval of 95%. Continuous variables will be recorded as the mean differences with 95% confidence interval. When identical method or unit of measure of the intervention effect in all studies is observed, weighted average difference is preferred. On the other hand, standard mean difference will be used to express the size of the same intervention effect in each study related to the variability observed in that study.

To explore the impact of the statistical heterogeneity on the meta-analysis, we will primarily use forest plots to assess any sign of potential heterogeneity visually. Then *I*^2^ statistic and Chi-squared test will be used to assess the presence of statistical heterogeneity and calculate the heterogeneity severity. When *P* > .1, *I*^*2*^ < 50%, it is considered that there is acceptable heterogeneity between the trials, and the fixed effect model will be chosen;^[25,26]^ otherwise, the random effect model will be considered and we will try to explain the underlying cause of heterogeneity by subgroup or sensitivity analysis (see “Subgroup analysis and sensitivity analysis” section below).^[27]^ If heterogeneity is high, the meta-analysis should be avoided.

#### 2.7.2 Subgroup analysis

When heterogeneity is identified, we will apply subgroup analysis to explore the source of heterogeneity. As the first step in the analysis of subgroup, make sure that the number of studies is adequate. Then we will stratify the subgroup by different subdomains. The criteria are as follows: population characteristics, complications, research quality, type of control interventions, dose and duration of intervention, follow-up period.

Moreover, we will also perform analysis for any other subgroups as reported in the included studies. It should be noted that selective reporting bias should be paid attention and minimized.

#### 2.7.3 Sensitivity analysis

To check the robustness of pooled data of the review process, we will perform sensitivity analysis. The principle decision nodes included methodological quality, the effect of missing data, type of study, sample size. We will delete every eligible study 1 by 1 and a second meta-analysis will be performed. The result will be compared and discussed in order to test whether the results could have been influenced by a single study.

### 2.8 Reporting bias

As most publications process tends to favor positive results or large-scale studies, the meta-analysis that aims to pool and analyze prepublished studies should be examined for the presence of any publication bias. We will use funnel plots, Egger test and Begg tests to assess the publication bias if more than 10 of the studies are included.^[28,29]^ Egger bias indicator test will be used to plot a regression line, which shows the symmetry of the plotted studies and examine for the presence of any publication bias. If insufficient number of articles are included, the test for funnel plot asymmetry will be inappropriate.^[30]^ If publication bias is significant, trim and fill method will be constructed for correcting the probable publication bias.

### 2.9 Confidence in cumulative evidence

The strength of evidence will be assessed on the Grading of recommendations, assessment, development, and evaluation system according to eight criteria: indirectness, inconsistency (heterogeneity), imprecision, and/or publication bias in addition to 4 criteria of risk of bias assessment tool.^[31]^ Quality of evidence for each outcome will be graded as high, moderate, low, or very low.

### 2.10 Ethics and dissemination

Research ethic approval will not be required because no individual patient data will be collected. We expect this systematic review will be published in a peer-reviewed journal.

## 3 Discussion

COVID-19 has been officially announced as a global pandemic, which are associated with a high rate of coagulopathy and high risk of VTE. Emerging evidence shows that anticoagulant therapy might improve the prognosis of severe COVID-19 patients. However, whether antiplatelet/anticoagulant therapy should be used in COVID-19 patients who required advanced life support until diagnostic assessment for VTE is still controversial. In addition to its safety and effectiveness, the current study also provides the evidence on the characteristics of therapy (eg, types, doses, frequencies and lengths) and participants. Therefore, we will summarize the current systematic analysis to figure out what the role of antiplatelet/anticoagulant therapy in prophylaxis of patients with severe COVID-19 is.

Currently, this is the first systematic review to evaluate the application of antiplatelet/anticoagulant therapy as preventive agents for patients with severe COVID-19. It will be reported following Cochrane Handbook for Systematic Reviews of Interventions and PRISMA statement guidelines. The potential limitations that inherent to systematic reviews and meta-analyses include publication bias, poor statically analyses and methodological quality, information bias, as well as inadequate reporting of methods and findings of the included studies. This review will provide valid advice and consultation for clinicians on the management of prophylactic antiplatelet/anticoagulant for severe COVID-19 patients.

## Acknowledgments

Not applicable.

## Author contributions

**Conceptualization:** Can Chen, Wei Hu.

**Data curation:** Yiwei Li, Ying Xu.

**Formal analysis:** Can Chen, Yiwei Li, Ying Xu.

**Funding acquisition:** Wei Hu.

**Methodology:** Can Chen, Yiwei Li.

**Project administration:** Can Chen, Wei Hu.

**Software:** Yiwei Li, Ying Xu.

**Supervision:** Can Chen, Wei Hu.

**Validation:** Yiwei Li, Ying Xu, Pengfei Shi, Ying Zhu, Can Chen, Wei Hu.

**Writing – original draft:** Yiwei Li, Ying Xu, Can Chen.

**Writing – review and editing:** Yiwei Li, Ying Xu, Pengfei Shi, Ying Zhu, Can Chen, Wei Hu.
